# Intraosseous Lipoma of the Calcaneus: A Systematic Review of Reported Cases, Imaging Characteristics, and Treatment Outcomes

**DOI:** 10.7759/cureus.99932

**Published:** 2025-12-23

**Authors:** Adam W Youssef, Paul M Doney, Cameron Ballard, Dryden Dalbey, Jayden Harrington

**Affiliations:** 1 College of Osteopathic Medicine, Kansas City University of Medicine and Biosciences, Joplin, USA

**Keywords:** benign bone lesion, bone tumor imaging, calcaneus, curettage, intraosseous lipoma, milgram staging

## Abstract

Intraosseous lipoma (IOL) of the calcaneus is a rare benign tumor composed of mature adipose tissue that may undergo fat necrosis and calcification. Because its imaging features can overlap with other radiolucent bone lesions, calcaneal IOL is sometimes misclassified and may prompt biopsy or surgical intervention for definitive diagnosis. To better characterize this entity, we conducted a systematic review following the Preferred Reporting Items for Systematic Reviews and Meta-Analyses (PRISMA) guidelines and performed a comprehensive PubMed search using the terms “intraosseous lipoma”, “calcaneus”, and “heel bone”. Eligible studies included case reports and series published between January 1976 and May 2024 that confirmed IOL of the calcaneus through histopathology. A total of 27 studies met criteria, representing 36 patients with a mean age of 40.3 years and a male predominance of 58.3%. Most cases were discovered incidentally or presented with mild heel pain. Imaging typically demonstrated well-defined lytic lesions with central calcification or fat attenuation, and histopathologic descriptions generally aligned with Milgram Stage I or II, though the staging system was not consistently reported. Surgical curettage was performed in 72.2% of patients, while 27.8% were managed conservatively. No malignant transformation or recurrence was reported. Calcaneal IOL should remain in the differential diagnosis for radiolucent heel lesions, and awareness of its characteristic imaging features may reduce unnecessary invasive procedures. While surgery is often chosen for symptomatic cases, conservative management is reasonable for incidental findings. Further research is needed to establish standardized diagnostic and treatment guidelines for this uncommon lesion.

## Introduction and background

Intraosseous lipoma (IOL) is an uncommon benign tumor composed primarily of mature adipocytes within the bone marrow cavity [1]. Accounting for less than 0.1-2.5% of all primary bone tumors, IOL is most frequently found in the calcaneus, femur, and tibia, with the calcaneus being a particularly favored site due to its abundant fatty marrow [2]. While often asymptomatic and discovered incidentally on imaging, some patients present with nonspecific heel pain or discomfort, prompting further diagnostic workup [3].

Radiographically, IOLs may resemble other lytic lesions, including unicameral bone cysts, aneurysmal bone cysts, or even malignancies, which can lead to diagnostic uncertainty. Given its rarity and often indolent course, the diagnosis and management of calcaneal IOLs are not standardized. Some cases are managed conservatively with observation, while others undergo surgical curettage due to persistent symptoms or unclear radiographic findings. To date, no comprehensive synthesis has focused specifically on calcaneal IOLs, despite multiple case reports and small series in the literature [4,5].

The purpose of this systematic review is to consolidate all published cases of IOL of the calcaneus, highlighting patterns in diagnosis, imaging findings, treatment strategies, and clinical outcomes. Through this analysis, we aim to clarify the natural history of this lesion and support more evidence-informed clinical decision-making.

## Review

Methods

Study Design and Objective

**Figure 1:**
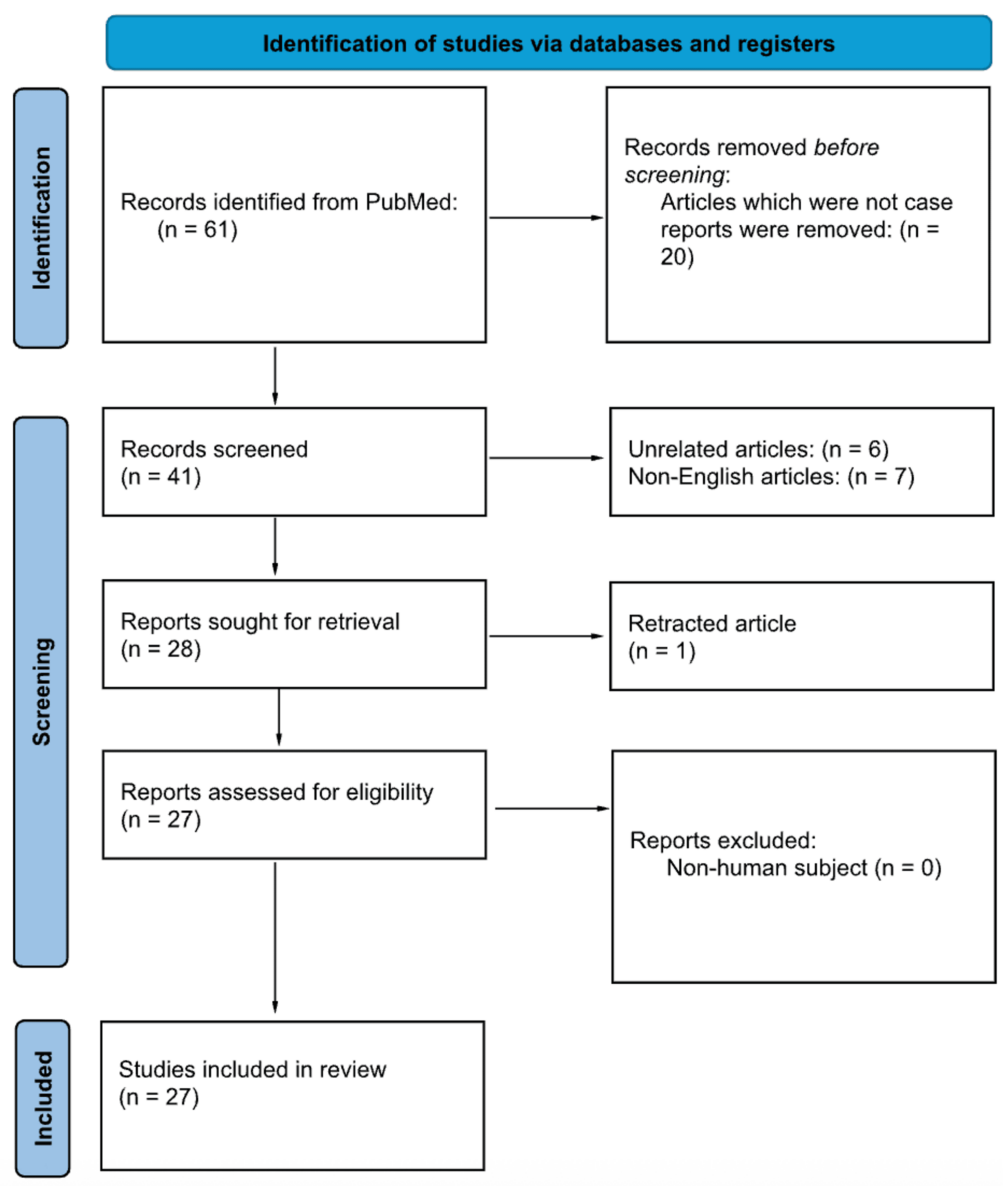
PRISMA flow diagram. Flow of records through the systematic review process: records identified, screened, assessed for eligibility, and included in the final dataset. PRISMA: Preferred Reporting Items for Systematic Reviews and Meta-Analyses

This study was conducted as a systematic review in accordance with the Preferred Reporting Items for Systematic Reviews and Meta-Analyses (PRISMA) guidelines (Figure 1). The objective was to synthesize all English-language case reports and case series published between January 1976 and May 2024 that described the clinical presentation, imaging features, management, and outcomes of IOL of the calcaneus. Given the rarity of this condition, only case-based literature was eligible for inclusion. The final PubMed search was completed in May 2024, and no additional databases (e.g., Embase or Scopus) were searched. Titles and abstracts were independently screened by two reviewers, followed by full-text review for eligibility, with discrepancies resolved by consensus.

Search Strategy

We conducted a comprehensive search of published case reports and series in PubMed without time restrictions, though only English-language full-text reports were included. The search terms involved were “intraosseous lipoma”, “calcaneus”, and “heel bone”. The specific search strategy can be seen in Table 1.

**Table 1:** Search strategy diagram. Search terms and Boolean operators used to identify cases of intraosseous lipoma of the calcaneus from the literature.

Serial #	Search Terms
#1	("intraosseous lipoma"[Title/Abstract])
#2	("calcaneus"[Title/Abstract] OR "heel bone"[Title/Abstract])
#3	#1 AND #2
Final Combined Search	(("intraosseous lipoma"[Title/Abstract])) AND (("calcaneus"[Title/Abstract] OR "heel bone"[Title/Abstract]))

Inclusion Criteria

In this study, English-language full-text case reports and case series on human subjects were eligible. Articles had to describe the clinical presentation, diagnosis, or treatment of an IOL of the calcaneus.

Exclusion Criteria

Non-human studies, letters to the editor, studies unrelated to IOL of the calcaneus diagnosis or management, and abstracts without full-text availability were excluded. Studies failing to meet quality or relevance thresholds were also omitted to ensure methodological rigor.

Outcome Measures

The primary outcomes assessed in this systematic review were symptom resolution, surgical versus non-surgical intervention, and malignant transformation. Outcomes were extracted from each included study based on the reported clinical follow-up, imaging assessments, and intraoperative findings. 

Data Extraction and Analysis

Study selection followed a two-step screening process. Initially, titles and abstracts were reviewed to assess relevance. Articles that met the inclusion criteria then underwent full-text review to confirm eligibility. The selection process is outlined in the PRISMA flow diagram (Figure 1). Data extraction was performed systematically using a standardized collection form to ensure consistency and completeness. The extracted information included the following: (1) key bibliographic details (author(s) and year of publication); (2) patient-specific data (age, sex); (3) diagnostic findings (clinical presentation, diagnostic methodology, histopathology, and imaging findings)); (4) treatment data (surgical versus conservative management, duration); and (5) outcomes (recurrence, resolution, malignant transformation). A summary of the extracted variables is provided in Table 2.

**Table 2:** Data extraction summary. Comprehensive overview of the reported demographic characteristics, imaging features, treatment approaches, and clinical outcomes for 36 cases of intraosseous lipoma of the calcaneus. Corresponding references for all included studies are listed in the References section.

Author (Year)	Patient Demographics: Age, Sex	Clinical Presentation	Diagnosis: Imaging Modalities (CT, X-ray, MRI), Histopathology	Treatment: Surgical vs Conservative Management, Duration	Outcomes: Recurrence, Resolution, Malignant Transformation
Schuh et al. (2022)	49 y/M	Plantar heel pain for 3 months. No trauma history. Tenderness near the plantar fascia	X-ray: Lytic lesion with bulls-eye ossification CT: Fat-density cystic lesion	Conservative management	Patient asymptomatic at follow-up
Yadav et al. (2024)	35 y/F	1-year history of heel pain and tenderness over the lateral heel. Worsened by uneven surfaces. No trauma	X-ray: cystic lesion MRI: suggestive of intraosseous lipoma	Surgical intervention	Complete resolution at 2.5-year follow-up
Aumar et al. (2013)	68 y/M	Heel and leg pain for 2 months, exacerbated with walking. Mild swelling. No history of trauma	X-ray: 2.0 x 2.5 cm cystic lesion with central sclerotic nidus CT: Well-circumscribed lesion with central calcification Histopathology: Mature adipose tissue with thin, degenerated trabeculae	Surgical intervention	Returned to normal activities after 15 months. Slight tenderness at incision, no recurrence. Imaging showed bone replacement of the lesion
Powell et al. (2018)	18 y/M	9/10 leg pain during basketball. Later improved and became asymptomatic over time. Lesion followed for 4 years	Initial X-ray: lytic lesion MRI: bilobed cyst with a narrow neck to the subtalar joint, gradual peripheral fat deposition consistent with developing lipoma	Conservative management–serial MRI over 4 years	The lesion matured into an intraosseous lipoma, however, the patient remained asymptomatic. Radiologic follow-up showed a stable lesion with a peripheral fat rim
Malghem et al. (2017)	15 y/M	Lesion discovered incidentally	Initial CT: 25 HU fluid-density cavity with mild expansion MRI: low T1 / high T2 signal consistent with simple bone cyst	Conservative management. Observation with 6- to 12-month imaging follow-up	Over 7 years, the cavity’s content converted from fluid to fatty marrow without change in size. No recurrence.
Hatori et al. (2001)	Case 1: 36 y/F Case 2: 22 y/F	Case 1: Heel pain Case 2: Heel pain, recurred 8 years after initial presentation	X-ray: Well-circumscribed radiolucent lesions CT: Fat-density masses MRI: High signal intensity on both T1- and T2-weighted images. Histopathology: Mature adipose tissue with small bone fragments, no atypia	Surgical intervention	Both patients had resolution of symptoms and no recurrence 2 years postoperatively
Weinfeld et al. (2002)	27 y/M (Total of 4 patients in this case report; however, only 1 patient presented with IOL of the calcaneus)	>5-year history of heel pain worsening over preceding months. Antalgic toe-gait. Plantar-heel tenderness	X-ray: Large lytic lesion without calcification MRI: T1 heterogeneous fat and hemorrhage Histopathology: mature adipose tissue with bone chips confirming intraosseous lipoma	Surgical intervention. Non-weight-bearing for 7 weeks, then progressive weight bearing	Full functional recovery by 9 weeks. No recurrence
Frangež et al. (2019)	44 y/F	18-month history of progressively worsening unilateral heel pain (VAS 10). Pain aggravated by high-heeled shoes	X-ray: Well-defined lytic lesion with calcification CT: Thin cortical shell and fatty density MRI: Fat-signal lesion with 1–2 mm cortex Histopathology: Mature adipose tissue consistent with intraosseous lipoma (Milgram II/III)	Surgical intervention	Full weight-bearing at 2 months. 12-month follow-up showed solid bone. No recurrence
Pappas et al. (2014)	38 y/M	Presented with a calcaneal fracture following minor trauma two days ago. Patient presents with intense pain, edema, and ecchymosis. Initially treated conservatively, later developed persistent pain and subtalar joint nonunion. Returned to the clinic after 6 months with pain.	X-rays and CT: Displaced calcaneal fracture MRI: High signal lesion on T1- and T2-weighted sequences Histopathology: Milgram Stage 1 intraosseous lipoma	Initial conservative treatment included non-weight-bearing activities and physical therapy. Surgical intervention afterwards.	Full resolution at 14 months. Imaging confirmed union and proper alignment. No recurrence
Sobota et al. (2024)	40 y/M	Incidental finding after ankle injury	X-ray: Well-defined osteolytic lesion MRI: Fat-containing lesion with a central mass showing contrast enhancement Histology: Adipose and smooth muscle cells	Biopsy followed by surgical intervention	Full recovery at 1.5-year follow-up
Greenspan et al. (1997)	Case 1: 57 y/F Case 2: 25 y/M Case 3: 67 y/F Case 4: 30 y/M Case 5: 25 y/M Case 6: 66 y/F	Case 1: Incidental finding during surgery for distal fibular fracture Case 2: Pain in the left hindfoot post-basketball injury, pain lasted 6 months Case 3: Intermittent left hindfoot pain for 12 months Case 4: >3 months pain of L calcaneus Case 5: < 3 months pain of R calcaneus Case 6: Incidental findings during imaging for unrelated foot injury	All lesions showed radiolucent areas with central ossification on radiographs Cases 1-3: Histopathologic confirmation of intraosseous lipoma Cases 4-6: Diagnosis based on plain radiographs, no histology provided	Cases 1-3: Surgical intervention Cases 4-6: Conservative management	Postoperative courses were uneventful for surgical patients. Non-surgical patients remained asymptomatic or stable over time. No recurrence reported
Karthik et al. (2011)	55 y/M	Two-month history of bilateral, non-traumatic heel pain. Worst with the first steps after rest and prolonged standing. Initially treated as plantar fasciitis	X-ray: Well-circumscribed lytic lesion with central calcification and thin sclerotic rim. CT: intact cortex around the lesion Histopathology: Mature adipose tissue with necrosis and irregular calcification (Milgram stage III)	Surgical intervention. Weight-bearing as tolerated	Complete symptom resolution. No recurrence
Narang et al. (2011)	38 y/M	Three-month history of dull, aching heel pain worsened by strenuous walking. Antalgic limp, toe-walking, and localized heel tenderness	X-ray: Well-circumscribed radiolucent lesion with trabeculations CT: cystic lesion with soft-tissue density, expanded inferior cortex, no cortical breach Histopathology: Mature lipocytes with medullary trabecular bone and areas of hemorrhage	Surgical intervention	Symptom resolution at 12-month follow-up. No recurrence
Poussa et al. (1976)	41 y/F	Lesion discovered incidentally on X-ray taken for vague bilateral ankle pain	Serial MRI at 4 and 7 years demonstrated progressive replacement of fluid by fat	Surgical intervention	No recurrence
Azarsina et al. (2019)	45 y/M	Chronic posterior right heel pain, worse in the morning and after long periods of inactivity. Unresponsive to 1 month of conservative treatment for presumed plantar fasciitis	X-ray: Unicameral lytic lesion with sclerotic margins and central calcification CT: Sclerotic border with minor cortical erosion MRI: Lesion with signal characteristics identical to fat. No evidence of plantar fasciitis Histopathology: Diagnosis confirmed intraoperatively and radiologically as intraosseous lipoma	Surgical intervention	Postoperative radiographs showed remodeling and healing of the graft site. No recurrence
Bertram et al. (2001)	38 y/F	Six-month history of heel pain increasing with weight-bearing	X-ray: Well-circumscribed lytic lesion with sclerotic margins MRI: Homogeneous high T1/T2 signal equivalent to fat and intact cortex Histopathology: Curettage specimen of mature adipose tissue with sparse bony trabeculae, confirming intraosseous lipoma	Surgical intervention	Symptom resolution by 12 months postoperatively. No recurrence
Balbouzis et al. (2019)	56 y/M	Three-month history of progressive heel pain	X-ray and CT: Well-defined hypodense cyst with central calcification MRI: Confirmed lipid content. Histopathology: Mature adipose tissue with sparse thin trabeculae, focal calcific necrosis, and no atypia	Surgical intervention	Symptom resolution at 6 months. No recurrence
Yip et al. (2024)	21 y/F	Two-month history of mild left heel pain, atraumatic. Initial management was observation	X-ray: Lucent lesion with sclerotic rim MRI: Central T2 hyperintensity with fluid and a small eccentric fat focus Follow-up MRI 3 yrs later: Homogeneous T1 hyperintense fat signal with complete fat suppression CT: Macroscopic fat. Histopathology: mature adipose tissue consistent with intraosseous lipoma	Conservative management	Lesion size and appearance remained stable on serial 6- to 12-month imaging. No recurrence
Gonzalez et al. (1997)	Case 1: 62 y/M Case 2: 31 y/F Case 3: 41 y/M	Case 1: Chronic left heel pain, initially misdiagnosed as plantar fasciitis Case 2: Severe right heel pain for 1 year Case 3: Left heel pain with an enlarging bony protuberance over several months	X-ray and CT for all cases. MRI was additionally used in Case 3. All diagnoses confirmed by histopathology showed adipose tissue consistent with intraosseous lipoma	Cases 1-3: Surgical intervention.	Case 1: No recurrence at 4.5 years Case 2: No recurrence at 2 years Case 3: Developed lateral nerve entrapment requiring two additional surgeries, no recurrence of lipoma at 2 years
Hassani et al. (2014)	26 y/F	Bilateral heel pain for 2 years	X-ray: Osteolytic lesions CT: Unicameral cyst-like and septated multicavitary lesion MRI: Fat density with calcifications Pathology: Foreign body granulomatous reaction with cholesterol clefts and multinucleated giant cells	Surgical intervention	No recurrence
Schatz et al. (1992)	45 y/M	Intermittent sharp ankle pain for 2 months	X-ray: Well-circumscribed lytic lesion with central sclerosis MRI: Fatty content with high signal intensity on T1 and T2 Histopathology: Mature adipose tissue with necrosis and calcifications	Surgical intervention	Asymptomatic at 15 months. No recurrence
Marberry et al. (2001)	Presumed adult	Heel pain	X-ray: Large lucent lesion with central calcification CT: Low-density lesion with calcific core Histology: Fibrofatty mass, confirmed intraosseous lipoma	Surgical intervention	Outcome beyond immediate surgery is not detailed
Tejero et al. (1999)	49 y/M	Ankle pain after mild torsional trauma	X-ray and CT: Well-defined osteolytic lesions with sclerotic borders Bilateral percutaneous biopsy confirmed intraosseous lipoma	Conservative management	No complications reported
Boylan et al. (1991)	39 y/F	Bilateral plantar heel pain for 1 year, worse with walking. Tenderness and mild edema on the right heel	X-ray: Well-demarcated radiolucent lesion with sclerotic rim and calcifications. CT: Low-density lesion Histology: Mature adipose tissue	Surgical intervention	At 11 months: full graft incorporation and resolution of symptoms
Ketyer et al. (1983)	Case 1: 52 y/M Case 2: 38 y/M	Case 1: Plantar foot pain Case 2: Incidental lesion during trauma workup	X-ray: Well-defined cystic lesion with central calcification CT: confirmed fat-density consistent with intraosseous lipoma	Conservative management	Unremarkable follow-up
Liapi-Avgeri et al. (1994)	35 y/M (Total of 3 patients in this case report; however, only 1 patient presented with IOL of the calcaneus)	Mild pain for > 3 months	X-ray: Radiolucent lesion with a thin sclerotic rim CT: Fat density and lytic changes. Histology: Mature adipose tissue. No fat necrosis or foamy histiocytes	Surgical intervention	No recurrence at 3-year follow-up
Gunterberg et al. (1978)	44 y/F	Right heel and leg pain for 5 months, worse with walking. Occasional lateral heel swelling	X-ray: Osteolytic, cyst-like lesion with central sclerotic area. Histopathology: Mature adipose tissue with regressive changes, foam cells, fibrosis, and calcification	Surgical intervention	Resolution at 13-month follow-up

Assessment of Bias

**Figure 2:**
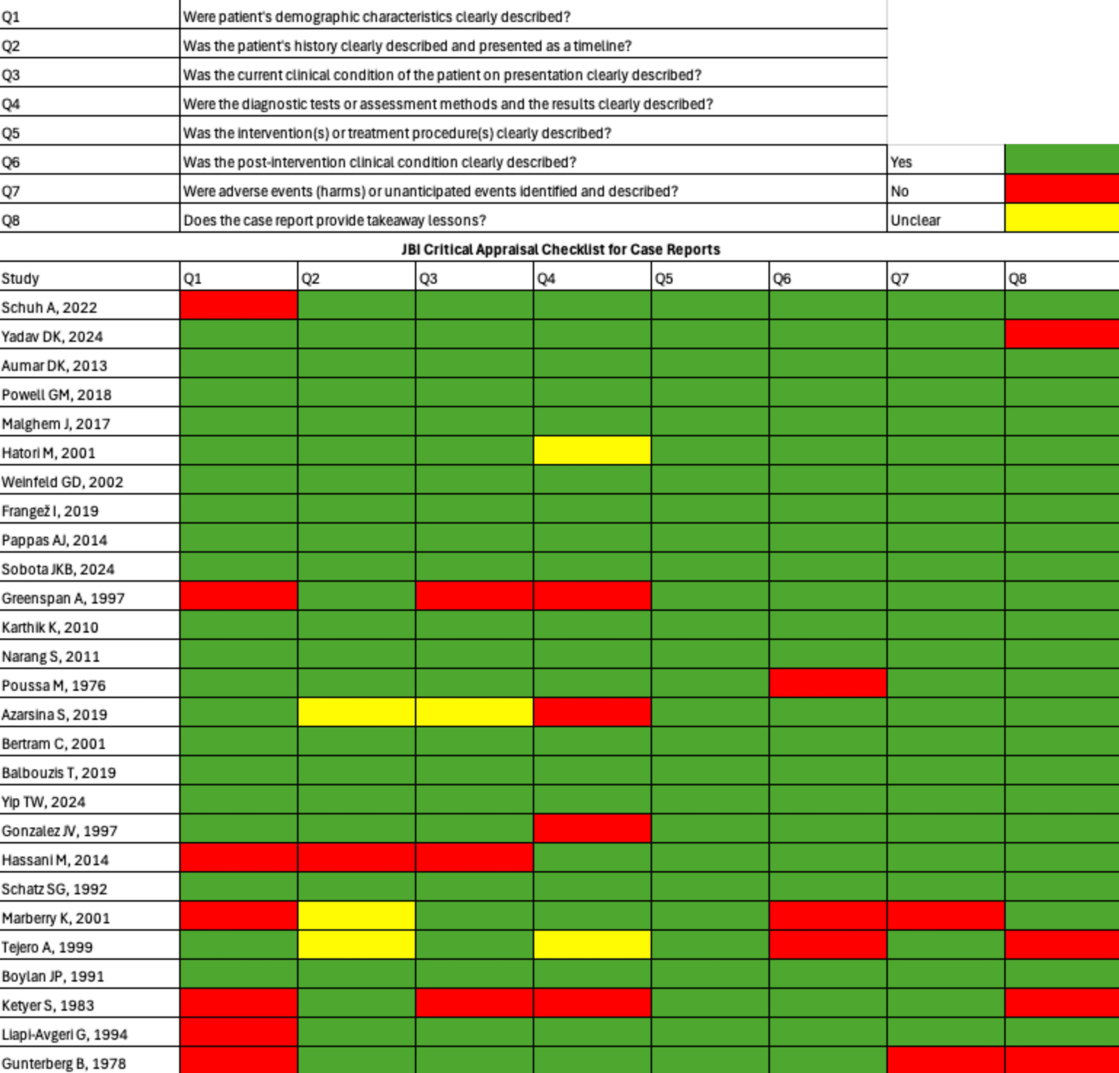
JBI critical appraisal summary. Assessment of the study quality using the Joanna Briggs Institute (JBI) Critical Appraisal Checklist for Case Reports and Case Series. Corresponding references for all included studies are listed in the References section.

We assessed the risk of bias using the Joanna Briggs Institute (JBI) Critical Appraisal Checklist [6]. This validated tool evaluates potential sources of bias and methodological limitations to determine the reliability of individual case studies. Each article was scored on an 8-point scale: scores of 7-8 were classified as high quality, 4-6 as moderate quality, and ≤3 as low quality. To ensure the integrity of this review, only studies rated as moderate or high quality were included. See Figure 2.

Results

Study Selection

A total of 61 articles were identified through the above PubMed search. After 20 articles that were not case reports or series were removed, the remaining 41 articles were subjected to title and abstract screening. Of these, 28 articles were relevant and abided by the inclusion criteria, and therefore underwent full-text review. Ultimately, 27 articles met the inclusion criteria and were included in this review [7-28]. The PRISMA flow diagram illustrates the detailed selection process, as shown in Figure 1.

Data Synthesis and Analysis

Due to the rarity of this condition, a meta-analysis was not feasible. Instead, a structured narrative synthesis was performed. Findings were grouped into four thematic domains: clinical presentation, diagnostic strategies, therapeutic approaches, and clinical outcomes. Recurring patterns and discrepancies were analyzed, with emphasis on the effectiveness and limitations of reported interventions. This approach also helped identify key knowledge gaps and directions for future research in the diagnosis and management of IOL of the calcaneus.

Characteristics of the Included Studies

All included publications were English-language case reports or small case series published between 1976 and 2024. Each study described a confirmed case of IOL involving the calcaneus, with varying degrees of clinical complexity and radiologic presentation. Despite being drawn from a diverse range of journals, all reports provided sufficient detail on clinical presentation, diagnostic imaging, treatment approach, and outcomes to meet the inclusion criteria.

Patient Demographics

Across the 27 included studies, data were available for 36 patients diagnosed with IOL of the calcaneus. The mean age at presentation was 40.3 years (range: 14-68), with a slight male predominance (58.3%). Additionally, a minority had comorbidities such as diabetes or Von Willebrand disease.

Clinical Presentation

The most common presenting symptom was chronic heel pain, reported in approximately 55.6% of cases. Pain was typically dull and insidious in onset, though some cases presented acutely following trauma or high-impact activity. Notably, 19.4% of lesions were discovered incidentally during imaging for unrelated complaints.

Imaging and Diagnostic Characteristics

Radiographically, IOLs most often appeared as well-circumscribed, radiolucent lesions with sclerotic borders and central calcification. X-ray was used in 86.1% of cases, computed tomography (CT) was used in 58.3% of cases, and magnetic resonance imaging (MRI) was used in 47.2% of cases. Histopathological confirmation was available in 66.7% of cases, most of which revealed mature adipose tissue consistent with lipoma. No malignant transformation of calcaneal IOLs was reported in the included cases.

Therapeutic Approaches

Of the 36 cases, 72.2% underwent surgical intervention, typically consisting of curettage with or without autologous or allograft bone grafting. Among surgically treated patients, autologous iliac crest bone grafting was the most commonly used method, accounting for 19.4% of surgeries, though a few cases incorporated allografts (8.3%), synthetic bone substitutes (e.g., β-tricalcium phosphate, Pro-Dense, chronOS Inject) (5.6%), or polymethylmethacrylate (PMMA) cement for structural support (2.8%). Prophylactic internal fixation was selectively utilized in patients with perceived high fracture risk based on clinical and radiographic features (e.g., pain, lesion size, and cortical involvement), rather than application of a validated scoring system. Postoperative protocols typically included a period of non-weight-bearing followed by a gradual return to full activity. Notably, patients managed conservatively showed stable or improving lesions on follow-up imaging, reinforcing the feasibility of observation in asymptomatic or minimally symptomatic cases.

Clinical Outcomes

Among surgically treated patients, complete symptom resolution was reported in 100% of cases with available follow-up data. Patients typically returned to full weight-bearing within six to nine weeks postoperatively, with many resuming normal activities and work within two to three months. Radiographic follow-up commonly showed successful graft incorporation, remodeling of the calcaneus, and no signs of lesion recurrence. No malignant transformations were observed across any of the surgically treated cases.

For conservatively managed patients, outcomes were similarly favorable. These individuals remained asymptomatic or reported stable, non-progressive lesions on serial imaging over extended follow-up periods - ranging from several months to as long as seven years. In at least one case, a fluid-filled lesion was observed to evolve into a fat-filled lipomatous lesion without surgical intervention or clinical deterioration.

Overall, no cases reported recurrence or malignant transformation, regardless of management strategy. The follow-up duration across all included cases ranged from six months to seven years, with several studies extending beyond the one-year mark, providing reassurance about the long-term stability and benign nature of IOLs of the calcaneus.

Discussion

IOL of the calcaneus, while rare, presents as a diagnostic challenge due to its radiographic resemblance to both benign and malignant bone lesions [1,2]. This systematic review analyzed 36 cases of histologically or radiographically confirmed calcaneal IOL to identify patterns in clinical presentation, imaging, and treatment.

Our findings demonstrate that most patients were adults who presented either with non-specific heel pain or with lesions incidentally discovered during imaging for unrelated complaints. Milgram classified IOLs into three stages based on histopathologic progression: Stage I lesions consist of viable mature adipose tissue without necrosis or calcification; Stage II lesions demonstrate partial fat necrosis with focal calcification; and Stage III lesions show advanced fat necrosis with extensive calcification, cystic change, or sclerosis. In the present review, Milgram staging was inconsistently reported, and staging terminology was applied only when explicitly described in the original studies. Consistent with prior literature, some lesions demonstrated features compatible with Milgram Stage I or II, including viable adipocytes with minimal to moderate necrosis and focal calcification.

Management strategies varied: asymptomatic patients were typically observed with serial imaging, whereas symptomatic cases were treated with surgical curettage, often accompanied by bone grafting. No cases reported malignant transformation or lesion recurrence during follow-up periods ranging from six months to seven years. These outcomes reinforce the benign and stable nature of calcaneal IOL. Surgical decisions were often guided by symptom severity or diagnostic uncertainty rather than objective lesion progression. The calcaneus is a particularly predisposed site for IOLs due to its abundant fatty marrow and the mechanical stresses placed on the hindfoot, which may contribute to lesion development and symptom manifestation [2].

Despite general consistency in outcomes, the literature remains limited. Many case reports lacked long-term follow-up or standardized imaging protocols. Additionally, inconsistencies in the application of Milgram staging hindered comparative analysis. Publication bias may also skew the literature toward symptomatic or surgically managed cases, underrepresenting the true prevalence and behavior of conservatively managed lesions. Future studies should aim to establish standardized diagnostic criteria and treatment regimens to support evidence-based decision-making in clinical practice.

Limitations

This systematic review is subject to several limitations. First, the analysis is based entirely on case reports and small case series, which inherently carry a risk of publication bias. Second, heterogeneity in reporting, particularly in imaging descriptions, surgical versus conservative management, follow-up status, outcome assessment, and the use of the Milgram staging system, limited our ability to perform a meta-analysis. Third, long-term outcomes were frequently unreported, hindering conclusions about recurrence or delayed complications. Finally, our search was limited to articles published in English and indexed in PubMed, potentially excluding relevant cases from other languages or databases.

## Conclusions

Intraosseous lipoma of the calcaneus is a rare but benign lesion with highly characteristic imaging features. When accurately recognized, these features can help avoid unnecessary biopsy or surgical intervention. Surgical curettage with or without bone grafting remains an effective treatment for symptomatic or radiologically ambiguous cases, but conservative monitoring is sufficient in asymptomatic patients. The absence of malignant transformation or recurrence across reviewed cases highlights the generally indolent nature of this tumor. To improve diagnostic confidence and treatment standardization, future research should focus on larger case series with long-term follow-up and consistent application of Milgram staging and advanced imaging protocols.
